# Supplementary figures and images for: Crosstalk of Cellulose and Mannan Perception Pathways Leads to Inhibition of Cellulase Production in Several Filamentous Fungi
Source: mBio. 2019 Jul 2;10(4):e00277-19. doi: 10.1128/mBio.00277-19 (PMC6606794; doi:10.1128/mBio.00277-19)

Fig S1.

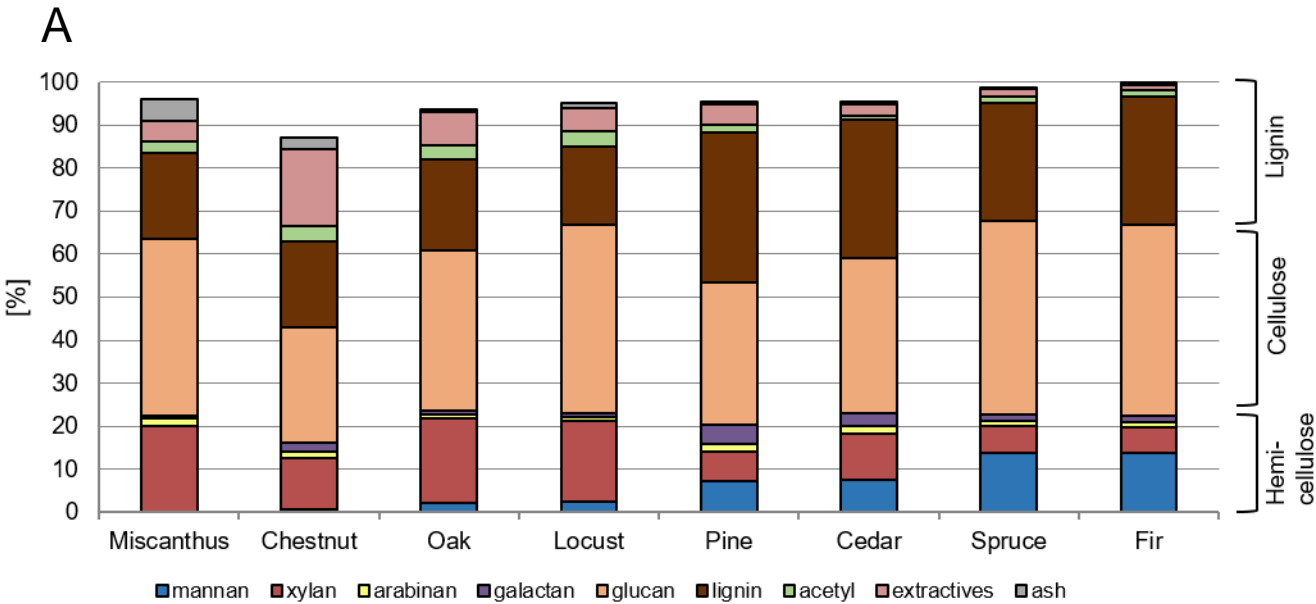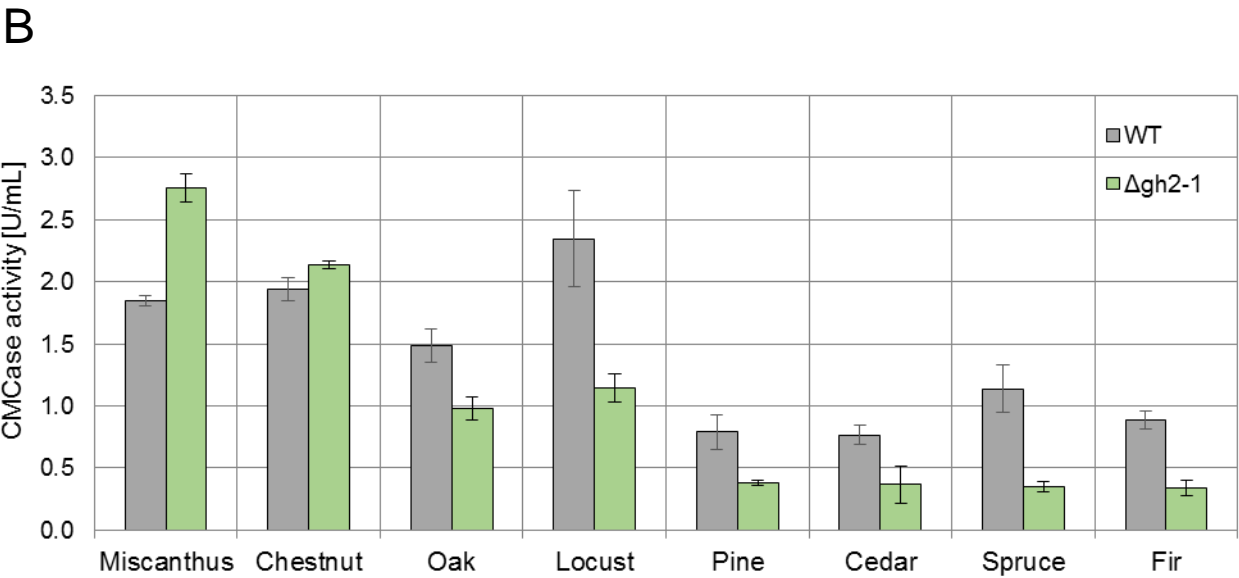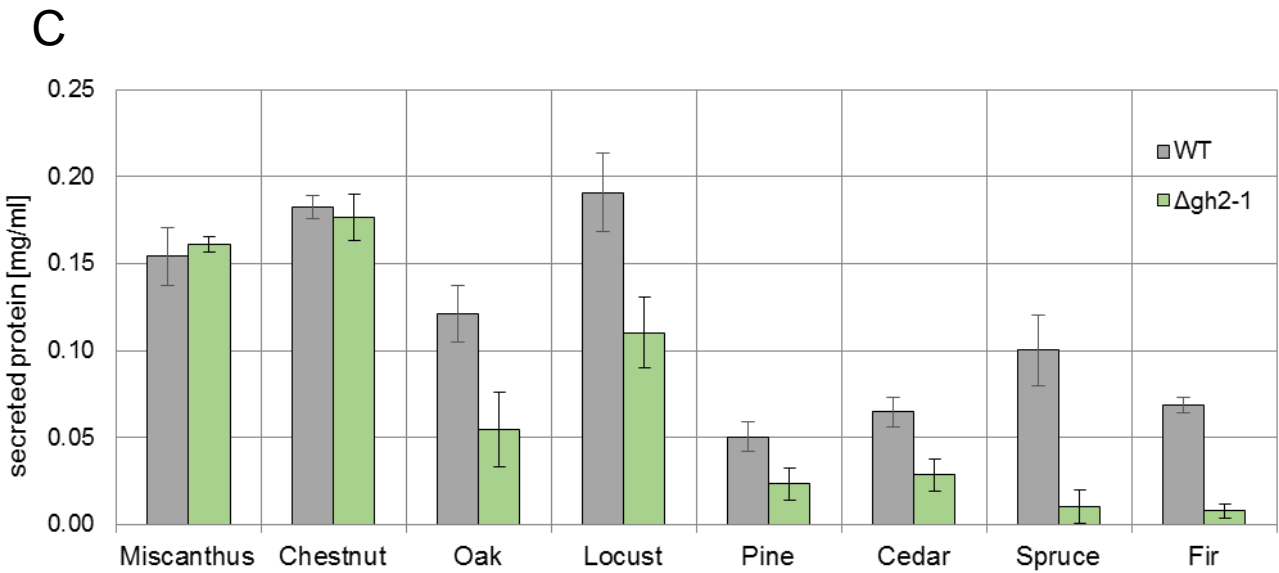

D

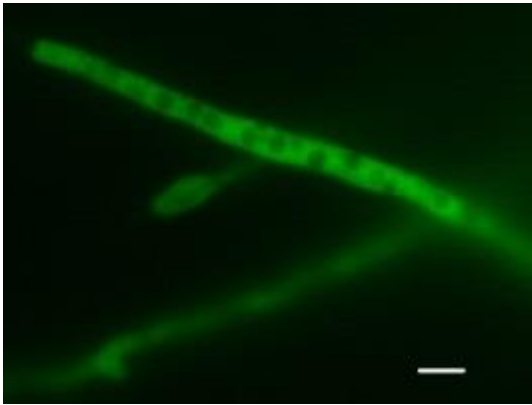

E

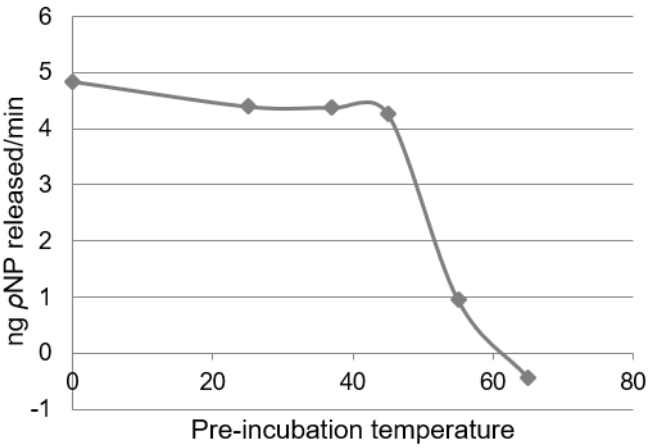

F

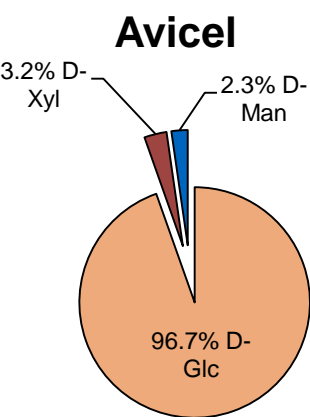

**Bacterial cellulose**

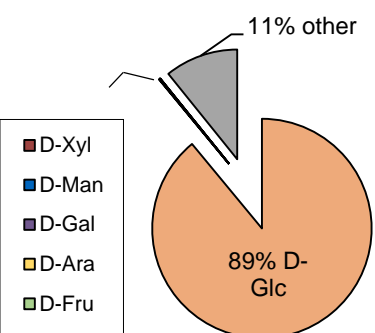

Supplement: FIG S1 [file mBio.00277-19-sf001.pdf]

Fig S2.

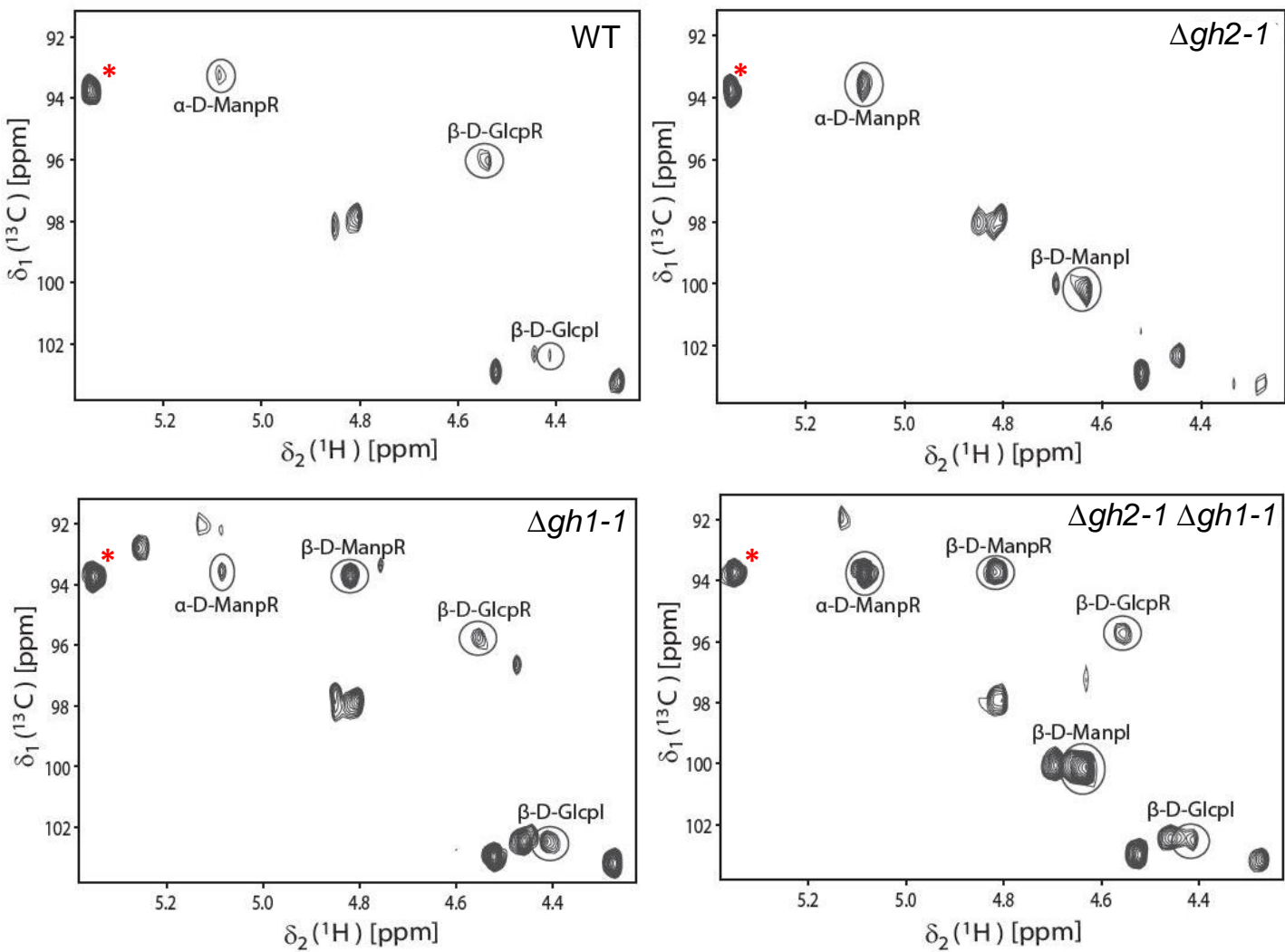

Supplement: FIG S2 [file mBio.00277-19-sf002.pdf]
