# Supplementary material for: Crosstalk of Cellulose and Mannan Perception Pathways Leads to Inhibition of Cellulase Production in Several Filamentous Fungi
Source: mBio. 2019 Jul 2;10(4):e00277-19. doi: 10.1128/mBio.00277-19 (PMC6606794; doi:10.1128/mBio.00277-19)
Supplement: TEXT S1 [file mBio.00277-19-s0001.docx]

# **Supporting Information**

## **Supplementary Methods**

### **Thermal stability assay**

The thermal stability assay was carried out by incubating the enzyme at different temperatures (0, 25, 37, 45, 55, and 65 °C) for 1 h. Afterwards, the residual activity of 0.1 µg enzyme was assayed with 50 mM KP buffer (pH 5.5) and 80 µg 4-Nitrophenyl-β-d-mannopyranoside substrate (Megazyme, Ireland, O-PNPBM). The reaction was incubated at 37 °C for 5 minutes, then stopped by the addition of 0.5 M Na_2_CO_3_ (pH 11.5). The absorbance was then measured at an OD of 405 nm.

### **Microscopy**

For gh2-1-gfp strain, the *gh2-1* gene amplified from gDNA was placed under the control of the constitutive promoter *ccg-1* (clock-controlled gene 1) using XbaI and BamHI restriction sites in plasmid pCCG-C-Gly-GFP. The construct was transformed into the WT *his-3* ^−^ strain by electrotransfection.

GFP fluorescence was visualized using an epifluorescence microscope with a 100x oil-immersion objective.
